# Novel Recycling of Epoxy Thermosets by Blending with Reversible Diels–Alder Epoxy Resin

**DOI:** 10.3390/polym16223205

**Published:** 2024-11-19

**Authors:** Isaac Lorero, Blanca Rico, Mónica Campo, Silvia G. Prolongo

**Affiliations:** 1 Materials Science and Engineering Area, Rey Juan Carlos University, C/Tulipán s/n, 28933 Móstoles, Madrid, Spain; b.rico.2016@alumnos.urjc.es; 2 Instituto de Investigación de Tecnologías para la Sostenibilidad, Rey Juan Carlos University, C/Tulipán s/n, 28933 Móstoles, Madrid, Spain

**Keywords:** Diels–Alder, thermosets recycling, CAN, thermo-reversible epoxy, thermosets blend

## Abstract

The introduction of Diels–Alder (D-A) bonds into epoxy resins is a promising pathway to convert these unrecyclable materials into sustainable materials. However, D-A bonds make epoxy resins extremely brittle materials and hinder their practical usability. Nonetheless, the reversibility of D-A bonds allows the transition of the material to a de-crosslinked network formed by separated oligomers that can melt above 90–100 °C. This means that D-A epoxy resins can be reprocessed after being cured like thermoplastics. In the present work, a thermoset blend is made by adding spent epoxy particles to a D-A epoxy resin to increase its thermal and mechanical properties and to evaluate a possible reuse of conventional thermoset wastes. The application of hot-pressing to a mixture of epoxy particles and powder of cured D-A epoxy creates a material in which the interaction of the particles with the D-A resin increases the thermal resistance of the material and prevents the D-A epoxy from melting at high temperatures. In addition, the flexural strength is increased by 80% and the chemical resistance against organic solvents is also improved.

## 1. Introduction

Over the past few decades, the use of thermosets has registered an upward trend in the industry, which implies some technological challenges once their life of service ends. Thermoset polymers usually offer moderate to low viscosities before curing and high adhesivity. After being cured, these materials acquire a highly crosslinked network, which gives them outstanding specific mechanical properties among polymer materials. Moreover, thermoset resins, such as epoxies, have other properties, like chemical resistance and low curing shrinkage, which make them suitable materials for coatings, insulators, or adhesive joints, among other industrial uses [1]. However, the formation of crosslinked networks with high thermomechanical properties and chemical inertness hinders the recyclability of thermosets [2,3]. Today, the use of oil for polymer synthesis demands around 10 million barrels of oil per day [4]. Within plastics manufacturing, thermosets already represent around 12%. The annual production of thermosets reaches around 44 million tons [5]; there numbers will grow from now to 2030 according to the forecast of the International Agency of Energy [4], even under policy scenarios of net zero emissions.

Conventional waste management of composites and thermosets is based on techniques unable to recover and reuse the polymer properly, such as landfilling or incinerating [2,6,7]. Landfilling is the least preferable option since it supposes the complete loss of wastes, an occupation of space, and severe environmental impact and health risks in the mid- and long-term [8]. Consequently, different government agencies, such as the European Union, are moving toward the complete restriction of this practice [9]. On the other hand, the combustion of thermoset wastes also supposes a total and irreversible loss of the material. Even though it mitigates the problem related to storage and recovers the notable calorific power of these materials to obtain thermal energy, this methodology is not exempt from disadvantages, especially those related to harmful and greenhouse gas emissions [5,7]. Beyond these conventional treatments, different thermochemical alternatives have been proposed over the last few years to give some recyclability to thermosets, such as pyrolysis [7,10,11,12], gasification [10] or solvolysis [2,5,7]. Pyrolysis and gasification treatments recover oils and gases suitable for energy uses by cracking the thermoset chains and some fractions of reusable monomers; meanwhile, solvolysis recycling achieves the recovery of reusable monomers and oligomers. On the other hand, these methods are not free of drawbacks: pyrolysis pathways, like other thermal treatments, generate noxious and greenhouse gas emissions, and solvolysis usually implies the management of hazardous liquid streams and certain complexities for large-scale implementation. Otherwise, thermoset wastes can also be treated through mechanical processes [2,5,11]. These processes are relatively simple and require moderate energy demands, but they suppose the loss of the original thermoset as structural material and downgrade its added value. Mechanical recycling is frequently based on cutting and grinding up to transform thermoset wastes into uniform flakes or small particles that are used as fillers for asphalt and concrete. Along these lines, the use of thermoset particles for thermoplastic matrices reinforcement is also under analysis [13,14,15], observing that they can increase certain thermal and mechanical properties similarly to the addition of other conventional fillers, such as some ceramic particles.

To overcome the disadvantages and difficulties of thermoset recycling, new approaches based on dynamic chemistry have been developed over the last decade, creating new covalently crosslinked networks with reversible bonds that give recyclability to thermosets [2]. These reversible thermosets, also known as covalent adaptable networks (CANs), switch and rearrange their structures through thermal stimuli. Depending on the exchange mechanism, CANs are grouped into two different categories: associative and dissociative. The associative CANs maintain a constant crosslink density across simultaneous exchange reactions when the temperature exceeds the recombination activation (T_v_) [16], using mechanisms such as transesterification [17], transamination [18], or imine [19] and disulfide [20] exchanges. Meanwhile, the dissociative CANs disengage the reversible bonds first and transit to a temporary fully loose structure analogous to a thermoplastic. Afterwards, a rearranged network can be built through a new crosslink reaction [21]. Most of the dissociative CANs are based on Diels–Alder (D-A) reactions. D-A reactions are cycloadditions between conjugated dienes and dienophiles, in which four π-electrons of the conjugated dienes and two π-electrons of the dienophiles react and generate σ-bonds, which are energetically more stable. As a result, unsaturated rings are formed. The introduction of switchable D-A crosslinks into thermosets to create dissociative CANs by adding furans (FAs) as dienes, and maleimides (BMIs) as dienophiles, is a field that has been under development in recent years [22,23,24,25,26]. Through activating this switchable structure, D-A resins can be recycled and thermoformed successfully by applying thermal and mechanical stresses [27,28,29,30]. One of the most common recycling treatments for CANs consists of milling, as in the mechanical recycling of conventional thermosets, followed by powder hot-pressing. As a result, a new solid bulk is rebuilt, and even more, it can show slight improvements in the mechanical and thermal properties compared to the original resin if the compaction is correctly optimized [27,30].

However, despite these exceptional advantages related to macromolecular mobility, D-A networks also have notable brittleness and a lack of mechanical properties in a glassy state compared with conventional thermosets [26]. D-A bonds are longer [31] and thermodynamically weaker [32] than single covalent bonds, which makes them more susceptible to mechanical breakage. This weakness becomes especially noticeable when the D-A bonds constitute all or a majority fraction of the thermoset network. For example, fully reversible D-A epoxies without permanent crosslinks have high brittleness and strengths below 10 MPa [24,26,33], which make them impractical, in contrast with the mechanical strength of conventional epoxies [24,26,34]. In addition, the chemical resistance against common organic solvents (dimethylformamide, dimethyl sulfoxide, etc.) is also low [24,33,35]. Nonetheless, the poor thermomechanical properties and fluidity of D-A epoxies, when the temperature reaches the activation of the retro D-A reaction (from 90 to 180 °C [29,33]), opens up the possibility of exploring the manufacturing of thermoset composites by adding fillers into previously cured D-A resins, as in the case of thermoplastic matrices’ reinforcement previously mentioned [13,14,15]. Thus, the possibility of enhancing D-A resin’s properties by recycling conventional thermoset wastes seems a synergic pathway to enhance the usability of these thermo-reversible resins and the circularity of thermosetting materials.

In the present work, the manufacturing of a D-A epoxy resin filled with conventional and non-reversible epoxy particles is proposed for the first time with the aim of initiating a new route for thermoset recycling joined with the development of new recyclable epoxy formulations based on dynamic bonds. The material is made by milling a conventional crosslinked epoxy resin with a fully reversible D-A epoxy resin. As a result, a mixture of non-dynamic thermoset particles and D-A epoxy powder is obtained and pressed at 130 °C and 150 bar to obtain a solid bulk. The obtained material shows higher thermal, mechanical, and chemical resistance in comparison with an unreinforced D-A epoxy resin due to the interaction among the bulk constituents.

## 2. Experimental

### 2.1. Materials

The reagents used for the resin manufacturing were the following: bisphenol A diglycidyl ether (DGEBA), m-xylylene diamine (MXDA), furfurylamine (FA), and 1,1′-(methylenedi-4,1-phenylene) bismaleimide (BMI). All the components were purchased from Merck (Darmstadt, Germany) and used as received.

**Figure 1:**
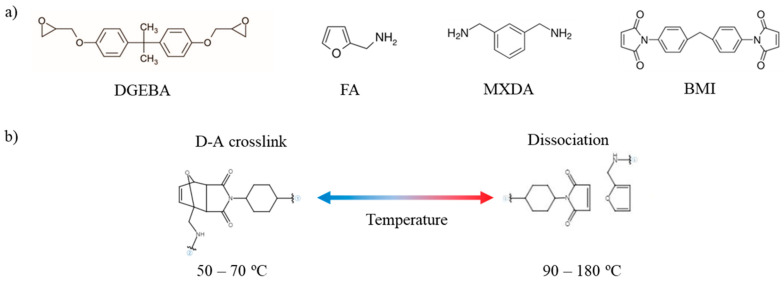
Structural formulas of DGEBA, FA, MXDA, and BMI (**a**), and reversible D-A bond formation and dissociation depending on the temperature applied (**b**).

Figure 1a shows the structural formulas of DGEBA, MXDA, FA, and BMI. The formation of D-A crosslinks is performed among furan groups of FA and maleimide groups of BMI. These crosslinks can be arranged from 50 to 70 °C, approximately, and disengaged when the temperature of the material is raised above 90 °C (Figure 1b).

To obtain a dissociative epoxy resin, the curing method has to be carefully set in terms of the temperature in order to minimize side reactions such as Michael addition, a non-reversible reaction between maleimides and amines that can impede the formation of the epoxy covalent network and the formation of D-A bonds. The manufacturing method used to allow the correct synthesis of the reversible resin, as developed in a previous work [26], is detailed in the next section.

### 2.2. Manufacturing

The initial resins were made according to a method developed in a previous work [26]. Specifically, the composition used in this work corresponds to a 0 D-A (DGEBA-MXDA) ratio and a 1.0 D-A ratio (DGEBA-FA-BMI). The adjustment of the reagent masses was performed according to the following equations:

(1) $$m_{FA}=\frac{m_{DGEBA}\cdot {Neq}_{DGEBA}}{{Mw}_{DGEBA}}\cdot \frac{{Mw}_{FA}}{{Neq}_{FA}}\cdot {DA}_{ratio}$$

(2) $$m_{BMI}=\frac{m_{FA}}{{Mw}_{FA}}\cdot {Mw}_{BMI}\cdot \frac{1}{2}$$

(3) $$m_{MXDA}=\frac{m_{DGEBA}\cdot {Neq}_{DGEBA}}{{Mw}_{DGEBA}}\cdot \frac{{Mw}_{MXDA}}{{Neq}_{MXDA}}\cdot (1-{DA}_{ratio})$$

where $m_{DGEBA}$, $m_{FA}$, $m_{BMI}$ and $m_{MXDA}$ are the masses of the epoxy monomer, furfurylamine, bismaleimide and m-xylylene diamine, ${Mw}_{DGEBA}$, ${Mw}_{FA}$, ${Mw}_{BMI}$ and ${Mw}_{MXDA}$ are the molar masses, ${Neq}_{DGEBA}$, ${Neq}_{FA}$ and ${Neq}_{MXDA}$ are the number of equivalents of epoxy per mole of DGEBA or amino equivalents per mole of furfurylamine and m-xylylene diamine, and ${DA}_{ratio}$ is the Diels–Alder bonds ratio of the resin.

To manufacture the traditional epoxy resin, 0 D-A ratio resin, DGEBA was heated up to 80 °C and degassed under mechanical stirring for 15 min. Then, MXDA was added, and the mixture was put in a mold to cure at 60 °C for 30 min and 100 °C for 6 h. On the other hand, to manufacture the totally reversible resin, 1.0 D-A resin, the reagents DGEBA and BMI were mixed and heated up to 80 °C with further mechanical stirring and degassing for 15 min. After, the reagents FA and MXDA were added, and the resin was molded to obtain the polymer. The curing cycles were set in two steps: first at a temperature of 100 °C for 6 h to generate covalent bonding and a second stage of 60 °C for 12 h to generate the Diels–Alder crosslinking [26].

After the curing of the resins, they were crushed to a fine powder in a miller and then mixed in proportions of 31 wt.% of 0 D-A ratio resin and 69 wt.% of 1.0 D-A ratio resin. These proportions give a blend with the same molar composition as a 0.6 D-A ratio resin whose properties and thermomechanical recycling through milling and hot-pressing were analyzed in previous works [26,30]. Finally, the powder mixture was put in a mold and hot-pressed at 130 °C and 150 bar for 30 min to create the recycled thermoset blend.

### 2.3. Characterization

Attenuated total reflectance–Fourier transform infrared spectroscopy (ATR-FTIR) was also used to analyze the chemical structure of the resins. For this purpose, scans from 500 to 4000 cm^−1^ were performed using a Nicolet iS50 from Thermo Fisher Scientific (Waltham, MA, USA).

Differential scanning calorimetry (DSC) tests were carried out with a DSC25 device from TA Instruments (New Castle, DE, USA) by setting a ramp from 30 to 240 °C with a 10 °C/min heating rate.

Thermal mechanical analysis (TMA) tests were applied using a Q400 machine from TA Instruments. The measurements were carried out for a temperature range from 30 °C to 200 °C, with a heating rate of 5 °C/min and an applied force on the samples of 0.02 N.

The thermomechanical properties were measured by dynamical thermomechanical analysis (DTMA) with a Q800 from TA Instruments. Tests were performed in single cantilever mode with an amplitude of 1% regarding the sample thickness and a frequency of 1 Hz. Thermomechanical data were collected in a temperature range from 30 °C to 150 °C, with a heating rate of 2 °C/min.

Flexure tests were carried out at ambient temperature on a universal testing machine Z100 from Zwick-Roell (Ulm, Germany), in the three-point bending mode using a 500 N load cell with a crosshead speed of 1.5 mm min^−1^, by ASTM D790. The fracture surfaces were sputtered with a thin layer of gold and observed via scanning electron microscopy (SEM) using a Hitachi S3400N (Tokyo, Japan) to investigate the morphologies.

The Vickers microhardness was measured by applying 1 kg loads (HV1) with an Innovatest Falcon 500 indentation tester (Maastricht, The Netherlands).

Wear tests were carried out at room temperature and under dry-sliding conditions with a ball-on-disc configuration using a Microtest SMT-A/0100 tribometer (Madrid, Spain). The wear tests were realized under 10 N loads and a sliding speed of 200 rpm, using steel balls. The tests were maintained up to a sliding distance of 1000 m. The samples were weighed before and after the wear tests to determine the mass loss and the wear tracks were observed with an optical microscope Leica DMR from Leica Microsystems (Wetzlar, Germany) equipped with Leica Image Pro plus software LAS V4.8.

The surface profilometries were determined using an SJ-301Surftest profilometer from Mitutoyo (Kawasaki, Japan).

## 3. Results and Discussion

**Figure 2:**
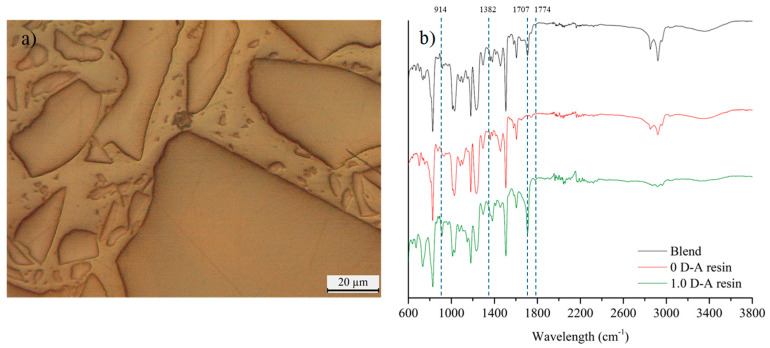
Image of the surface of the composite material in which the continuous reversible phase and the non-reversible resin particles are observed (**a**). Comparison of the infrared spectra of the blend with reversible and non-reversible resins (**b**).

The blend obtained from the hot compaction of the mixture of reversible (1.0 D-A ratio) and non-reversible thermoset particles (0 D-A ratio) shows a clear phase differentiation, as observable in Figure 2a. The reversible resin melts during hot compaction due to the retro D-A reaction, leaving the non-reversible resin particles dispersed throughout the material. The solid bulk is analyzed by infrared spectroscopy, avoiding those parts with coarse superficial particles of thermoset waste to not alter specific bands such as the C–N and C=O characteristic bands of maleimide groups. The analysis is carried out to corroborate that the thermomechanical treatment does not induce significant changes in the chemical structure. As shown in Figure 2b, there are no qualitative changes in comparison to the spectra of the constituent resins. The spectrum of the hot-pressed bulk shows the characteristic bands given by the presence of the bismaleimide. However, it has lower peak intensities than those observed in the infrared spectrum of the reversible resin. In this regard, the appearance of the characteristic bands of the C=O bond (maleimide groups, 1708 and 1774 cm^−1^) and the intensification of the C–N bond band (1382 cm^−1^) can be pointed out. In addition, there are also differences in the intensity of the 914 cm^−1^ band, which corresponds to unreacted epoxy groups and furfurylamine. The blend shows a less intense band compared to the reversible resin (1.0 D-A) due to the post-curing effect of hot-pressing that helps to increase the curing conversion and eliminates free remanent epoxy groups [30], and due to the presence of the non-reversible particles (0 D-A), which only show a faint peak at this wavelength. It is also worth noting the appearance of the -OH bond band between 3200 and 3600 cm^−1^ in the blend material after hot-pressing. In previous work, it was observed that the epoxy resins with D-A bonds show faint -OH bands due to the formation of hydrogen bonds with oxygen atoms of furans and nitrogen and carbonyl groups of maleimides [24,26]. Formation of -OH bonds seems reversible with heating, and D-A resins can show increased -OH bands in the FTIR spectra after being heated above 100 °C [26,30], as happens with the manufactured blend.

**Figure 3:**
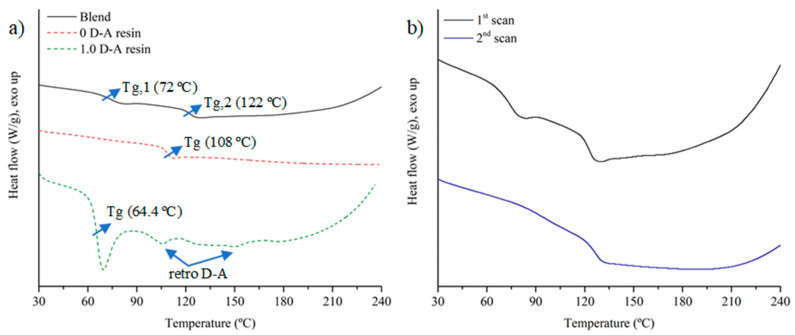
Comparison of the DSC scans of the recycled blend with 0 D-A and 1.0 D-A ratio resins (**a**), and comparison of the 1st DSC scan and 2nd DSC scan of the recycled blend from 30 to 240 °C (**b**).

Regarding the thermal response, differential calorimetry scans (Figure 3) reveal the composition of two phases with different behaviors in the blend, as is to be expected for a blend with two different polymer phases. Figure 3a compares the thermal behaviors of the blend and its constituents (0 D-A and 1.0 D-A ratio resin). The blend shows a first glass transition at 72 °C, followed by a second one at 122 °C, which correspond, respectively, to the reversible epoxy and the non-reversible particles. At higher temperatures, an exothermic curve above 180 °C can be observed due to the homopolymerization of the bismaleimide. This homopolymerization among maleimides confirms that the hot-pressing cycle does not activate this reaction or other irreversible reactions involving maleimide groups (such as Michael addition). The second glass transition at 122 °C overlaps with the retro D-A reaction, hindering the observation of the characteristic endothermic peaks made by reversible bond disengagement. Compared with its constituents, the blend scan shows two glass transitions that occur at temperatures higher than those observed in the cured 0 D-A and 1.0 D-A resins (64.4 and 108 °C, respectively). As mentioned in the FTIR analysis, hot-pressing induces post-curing in both phases that completes the reaction between the unreacted epoxy and amine groups after the initial curing, especially for the reversible resin. The increase in the glass transition temperatures agrees with the attenuation of the 914 cm^−1^ peak previously observed in the FTIR analysis.

Another interesting point is the effect of epoxy particles and hot-pressing post-curing on the reduction of thermal aging caused by the subsequent D-A reaction at 60 °C. As seen in Figure 3a, the blend does not show an enthalpy relaxation peak associated with the glass transition of the matrix that does appear in the DSC scan of the 1.0 ratio resin. Physical aging, caused by the proximity of the D-A heating temperature to the glass transition temperature of the 1.0 D-A ratio resin, is a reversible phenomenon [36] that can be attenuated or even eliminated if the resin is above the glass transition temperature for at least 10–15 min [37]. The hot-pressing process, performed with an isothermal and isobaric step at 130 °C for 30 min, can reverse the aging of the D-A resin. However, after the manufacturing of the solid bulk, the blend is heated again at 60 °C for 12 h to rebuild the Diels–Alder adduct network. The absence of the enthalpy relaxation of the DSC of the blend points to two possible effects. On the one hand, the post-curing caused by hot compaction raises the glass transition temperature of the reversible phase, which may mitigate aging by increasing the temperature gap with the D-A reaction temperature, thereby impeding configurational changes in the polymeric network. On the other hand, it could be possible that the presence of the ratio 0 resin particles dispersed in the ratio 1.0 matrix could also contribute to the attenuation of the physical aging. This type of behavior has been observed in some epoxy resin copolymers [37].

Finally, to complete the analysis, a second DSC scan (included in Figure 3b) after the first heating up to 240 °C is performed on the blend material. In this second scan, the slight endothermic peak of the dissociative phase upon reaching the glass transition disappears. At the same time, the area corresponding to the retro Diels–Alder reaction temperatures remains flat, and the exothermic slope above 200 °C that corresponds to the homopolymerization of the bismaleimide is much fainter. These aspects confirm the presence of both Diels–Alder and non-dynamic phases on the blend.

**Figure 4:**
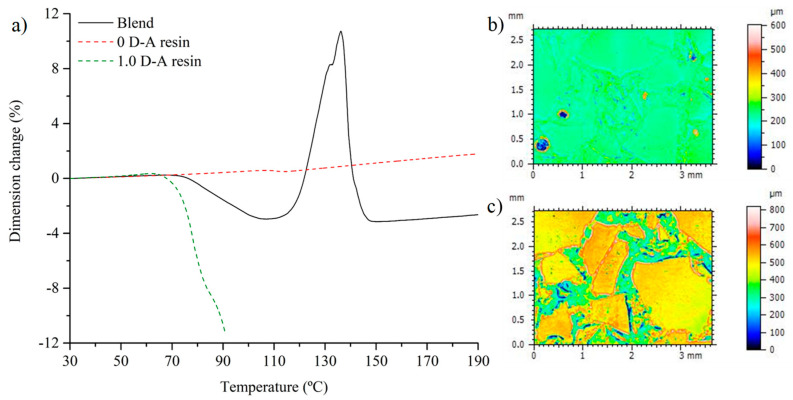
Comparison of the TMA curves of the composite material and the resins with Diels–Alder bond ratios of 0 and 1.0 (**a**). Topological image of the surface of the fabricated composite material (**b**) and after heating at 150 °C for 10 min (**c**).

Due to the presence of two phases in the blend material with different thermal behaviors, and considering that the reversible epoxy is meltable, it is decided to extend the analysis of the thermal behavior by including an evaluation of its dimensional stability with the temperature. Figure 4a shows the behavior of the 0 and 1.0 ratio resins and the blend material in the TMA test. While the non-reversible ratio resin shows the behavior expected in a conventional thermoset, the reversible epoxy softens abruptly with the glass transition and the onset of the retro Diels–Alder reaction, showing significant dimensional shrinkage under the compressive force exerted during the test. The test must be interrupted while the resin passes to a viscous liquid. The addition of 0 D-A ratio resin particles changes the thermal behavior significantly. Upon reaching the glass transition and the temperature of the retro Diels–Alder reaction, the blend shows a lower softening between 80 and 120 °C compared with the 1.0 D-A epoxy and does not melt. Nonetheless, the material loses dimensional stability above 120 °C due to the glass transition of the 0 D-A ratio particles. At this point, the particles change their thermal expansion and the cohesion between the phases is partially lost. Thus, the blend undergoes a strong volumetric expansion up to 140 °C. This is checked by performing surface profilometry on the material after its manufacturing and after heating it to 140 °C for 10 min. The observations are shown in Figure 4b,c: the material goes from having a flat surface to showing a relief surface with the reinforcement particles protruding upwards.

The increase in the thermal stability of a polymeric matrix through the introduction of thermoset particles was previously observed in the literature in those cases in which good interfacial adhesion is achieved [15,38]. The blend manufactured in the present work is a dissociable epoxy with a chemical composition that is quite similar to the non-dynamic epoxy particles. Both phases can contain small amounts of unreacted epoxy groups and free amines, which can bond to each other during hot compaction of the mixture and both generate the post-curing observed in the thermal characterization and enhance the matrix-reinforcement interaction, leading to the improved properties observed in the blend material.

**Figure 5:**
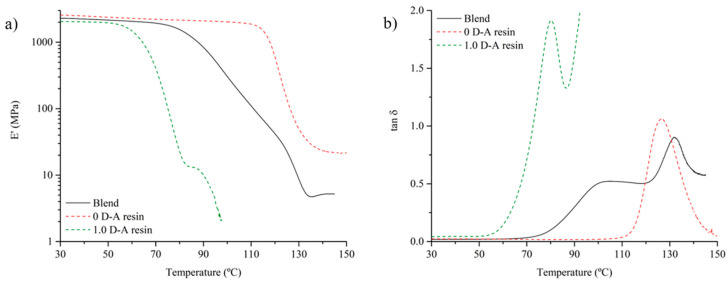
Comparison of the thermomechanical properties of the recycled thermoset blend with 0 D-A and 1.0 D-A ratio resins: evolution of the storage modulus (**a**) and tan delta (**b**) with the temperature.

The addition of non-reversible thermosetting particles to the Diels–Alder resin also has an analogous effect on the thermomechanical properties measured by DMTA (Figure 5). The blend material shows an intermediate response compared to its two constituents. Compared to the 1.0 D-A ratio resin, the blend shows some increase in the storage modulus (around 13% higher at 30 °C) due to the effective interaction among the phases and the post-curing caused by hot-pressing (Figure 5a and Table 1).

The blend initiates its glass transition with a decrease in E’ that starts at 83 °C, somewhat significantly higher than the temperature of initiation of the glass transition of the 1.0 D-A ratio epoxy resin (65 °C) [26]. In addition, the slope of the storage modulus decrease is less steep due to mobility restrictions exerted by the reinforcing particles. Once the temperature exceeds 124 °C, the glass transition of the non-reversible thermoset particles begins, and the slope changes again. A sudden drop in E’ is observed with the increase in temperature. At this point, the loss of cohesion between the constituents begins, and the test is interrupted. However, it is important to again note the absence of a melting point in the composite material, since after completing the glass transition, the material shows a rubbery plateau from 140 °C with storage modulus values near 5 MPa. Therefore, the addition of thermoset particles opens up the possibility of manufacturing thermo-reversible and recyclable matrices with enhanced thermal stability and thermomechanical properties, as well as avoiding their transition to a liquid state when a bond-reversing temperature is reached.

The thermomechanical behavior analyzed before can also be observed in the tan delta curves of the DMTA tests (Figure 5b). The addition of thermoset particles limits the D-A resin chain mobility, impeding resin flowing above the retro D-A reaction temperatures. Then, the tan delta values of the blend do not exceed values above 1. There can be observed a first relative maximum caused by the matrix glass transition at around 100 °C, which is nearly 20 °C more than the temperature of the maximum value of the tan delta of the non-reinforced 1.0 D-A resin (80.3 °C). Above 90 °C, the disengagement of the D-A crosslinks turns the fully reversible resin meltable [26], and then the tan delta values rise abruptly, something that does not happen in the blend samples. Above 120 °C, the glass transition of the non-reversible particles makes the tan delta values rise again, and then a maximum value of the tan delta in the blend material is reached at 133 °C, which is 6.5 °C higher than the tan delta maximum temperature of the 0 D-A ratio resin. The increase in the temperatures at which the tan delta maxima of the blend are reached in comparison with the 0 and 1.0 D-A resins again points to the post-curing effect of hot compaction.

**Figure 6:**
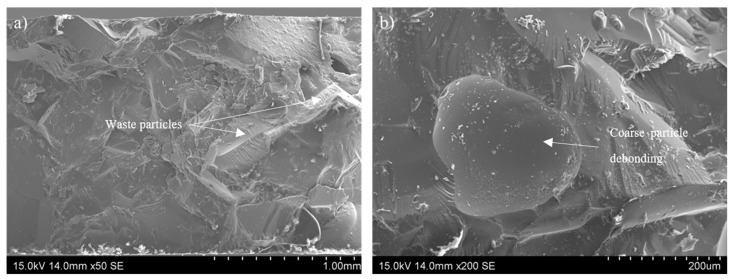
SEM observations of the three-point bending fracture of the recycled thermoset blend at low (**a**) and high magnifications (**b**).

The reinforcing effect of the thermosetting particles in the 1.0 D-A resin also influences the mechanical properties, as measured by three-point bending tests, and helps to increase their lower values (see Table 2). The blend has higher flexural stiffness (2.9 GPa) compared with the 1.0 D-A ratio resin without reinforcement (1.8 GPa), which indicates the formation of strong interfaces between the non-reversible thermoset particles and the reversible phase. The good interfacial adhesion also increases the three-point bending strength. The blend shows a strength that is 80% higher than those observed in the reversible resin (12.7 vs. 7 MPa); the break strain is also increased, being around 70% more than those observed in the dissociative resin without particles (1% vs. 0.6%). In this regard, Figure 6 shows a fracture of the blend material after the bending tests. The presence of smooth surfaces corresponding to interfaces between the particles and 1.0 D-A resin, in which crack propagation happens, is observed (Figure 6b). Nonetheless, the irregularity of the surfaces created by the presence of the thermostable particles contrasts with the brittle and totally smooth fracture surfaces characteristic of the unreinforced 1.0 D-A resin [26] (see Figure S1a,b), and it also contrasts with 0 D-A resins fractures (Figure S1c,d) that are more irregular due to its higher toughness.

Apart from the flexural tests, the effect of the addition of thermosetting particles on the hardness is also evaluated (see Table 2): the blend shows a slight decrease in this property. The average hardness is 20.4 HV, which is slightly lower in comparison with the 1.0 D-A epoxy resin (22.6 HV). In previous studies, it was observed that the D-A bond content did not significantly affect the epoxy resin hardness, with variations of 10–15% in the hardness as a maximum [26], which falls within the variation interval observed in the present research. In this regard, we did not find other studies in the bibliography with which to compare the possible effects on the hardness of epoxy particles’ addition to other epoxy matrix and to establish deeper conclusions.

The addition of non-reversible epoxy particles increases the wear strength notably. While the unreinforced reversible resin is not able to withstand the wear test conditions due to high mass loss and damage propagation, the blend does withstand the linear travel of 1000 m in contact with the steel ball under an applied load of 10 N and a rotational speed of 100 rpm. The blend shows a wear resistance similar to those observed in previous works in partially dissociative resins with D-A bond ratios between 0.2 and 0.4 [26]. Figure S2 shows this comparison of the wear resistance vs. the D-A ratio, which points out that the increase in the D-A bonds weakens the wear resistance, making epoxy resins with high ratios unable to resist wear test conditions.

**Figure 7:**
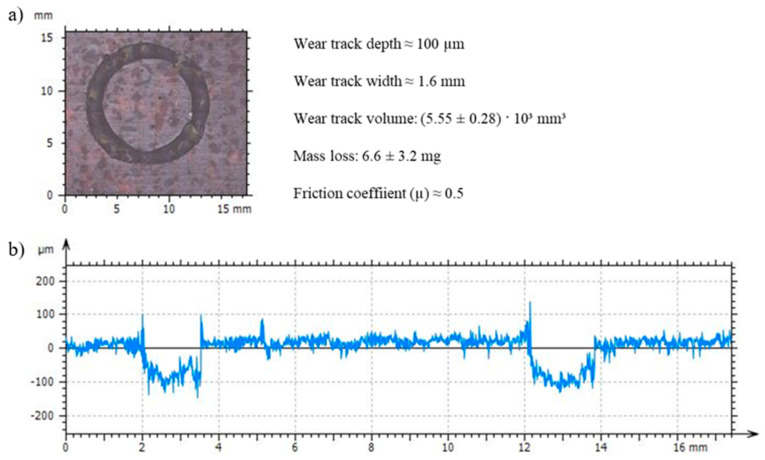
Observations of the recycled blend wear track under an optical microscope. The surface area with the wear footprint (**a**) and surface profile obtained by optical profilometry (**b**).

**Figure 8:**
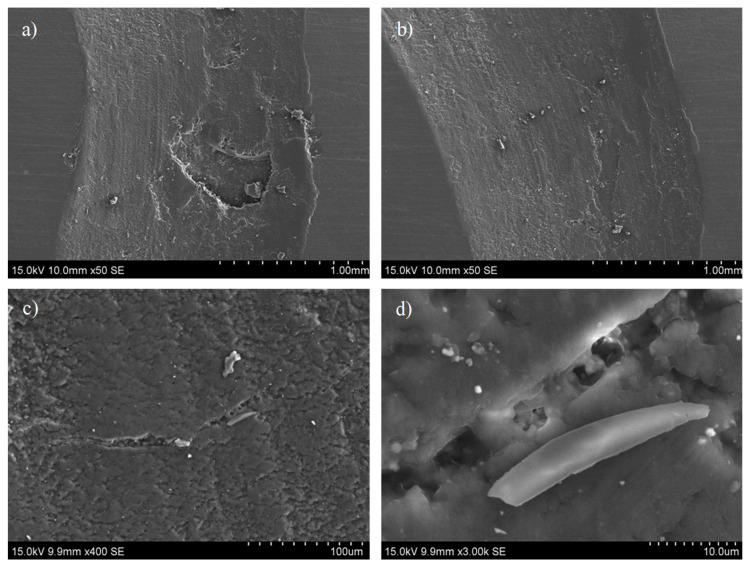
Observations of the recycled blend wear track in SEM at 50× (**a**,**b**) 400× (**c**), and 3000× (**d**).

The wear tracks of the recycled blend have approximately 100 µm of depth. The loss of mass during the tests varies slightly between 3 mg to 10 mg (see Figure 7). Crack nucleation in the recycled blend is still occurring. Nonetheless, the addition of the particles improves the wear resistance and its presence seems to slow down the fracture propagation due to the formation of strong interfaces with 1.0 D-A resin. In this regard, Figure 8 includes SEM observations of a wear footprint made in the recycled blend. The wear surfaces are certainly irregular due to debris formation and material removal phenomena (Figure 8a,b). The brittleness and low thermomechanical properties of the 1.0 D-A resin may lead to the formation of fine debris and its softening under wear conditions, and then to the adhesive wear tracks that can be observed in Figure 8c. In this figure, the apparition of cracks in the blend surface can also be observed. Figure 8d shows these cracks at high magnifications, in which some local unions appear due to the presence of particles covered by the reversible matrix.

**Figure 9:**
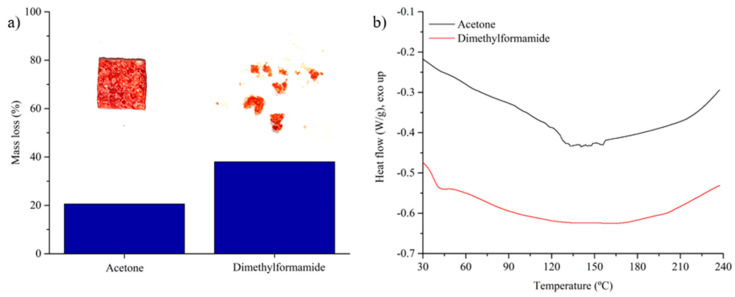
Mass loss of the thermoset blend after 24 h of immersion in acetone and DMF (**a**), and DSC scans of the recovered solids after the immersions (**b**).

Finally, the chemical resistance of the blend to exposure to organic solvents is tested. The material shows a notable improvement compared with the 1.0 D-A epoxy. Figure 9a shows a comparison of the mass losses after immersion in acetone and dimethylformamide at room temperature for 24 h. A comparison of the chemical resistance against these organic solvents of the epoxy resin vs. the D-A ratio is shown in Figure S3. In it, it can be observed that the addition of dissociative bonds makes the epoxy resins more susceptible to dissolving in organic solvents; something that is common in this type of material according to the studies published in the bibliography [24,33,35]. The constituents of the blend (0 and 1.0 D-A ratio resins) have opposite behaviors when exposed separately to the organic solvents used (acetone and dimethylformamide). The 0 D-A ratio resin, as a non-dynamic thermoset, is not affected by its exposure to these solvents. Meanwhile, the dissociative resin is fully dissolved in both reagents after 24 h of immersion.

Therefore, it would be expected to see a quasi-complete dissolution of the reversible phase (approximately 69% by weight of the material), recovering only separated particles of non-dynamic 0 D-A epoxy (around 31% by weight of the material). This, however, does not happen. In the case of immersion in acetone, after 24 h, the blend retains its continuity and most of the Diels–Alder phase. The samples show a mass loss near to 20% after being dried in a vacuum oven at 60 °C for 12 h to eliminate the organic solvent. When immersed in dimethylformamide, a more aggressive solvent, the blend loses its integrity and the loss of mass of the material is higher (near to 38%). After immersion and drying in DMF, small aggregates of non-dynamic epoxy particles and D-A resin are recovered.

The materials obtained from the solvent immersion and drying process are analyzed by differential scanning calorimetry. The results are shown in Figure 9b. The scans show the effect caused by the partial dissolution of the reversible phase. Above 200 °C, the materials trace exothermic heat flows caused by the triggering of the bismaleimide homopolymerization. In the case of the material immersed in acetone, due to the lower loss of D-A resin, the exothermic slope is more pronounced.

## 4. Conclusions

The possibility of improving the mechanical and thermal properties of the resin with a Diels–Alder bond ratio of 1.0 has been evaluated by adding reinforcements in the form of conventional thermostable particles. Based on the results obtained, the following conclusions can be drawn:

1. Hot compaction allows an effective obtention of materials composed of purely dissociative resins mixed with conventional thermoset particles.
2. Some mechanical properties of the 1.0 D-A ratio resin, such as the flexural strength and wear resistance, can be significantly improved by the addition of thermoset particles. The flexural strength increases from 7 MPa to 12.7 MPa, and the recycled blend is able to resist wear test conditions without breaking, which contrasts with the low properties of the unreinforced 1.0 D-A resin.
3. The hot-pressing method also generates certain interactions between the reversible and irreversible polymer networks and induces post-curing, improving the thermal properties and chemical resistance. The blend does not melt above 100 °C and presents certain resistance to its exposure to organic solvents, which contrasts with the thermal and chemical behavior of a purely dissociative epoxy resin.

In summary, this work develops a novel focus for thermoset waste recycling. The positive effect of its inclusion in D-A resins is demonstrated for future developments, in which the lack of properties of this type of recyclable resins can be ameliorated. Alternative strategies, such as the addition of different weight contents of particles and different thermosetting waste materials, not only in cured hot-pressed D-A resins but also in liquid D-A resins before carrying out the curing cycle, can be proposed.

## Figures and Tables

**Table 1:** Thermomechanical properties comparison of the thermoset blend, 0 D-A, and 1.0 D-A ratio resins.

	Blend	0 D-A Ratio Resin	1.0 D-A Ratio Resin
E′_30 °C_ (MPa)	2176 ± 185	2656 ± 146	1930 ± 74
E″_max_ (MPa)	217.2 ± 48.3	329.3 ± 14.3	287.1 ± 8.5
T_g, max tan δ_ (°C)	133 ± 2.1	126.5 ± 0.3	80.3 ± 1.9

**Table 2:** Comparison of the mechanical properties between the reversible resin and the blend with non-reversible particles.

	1.0 D-A Ratio Resin	Blend
E (MPa)	1788 ± 24	2915 ± 90
σ (MPa)	7 ± 1	12.7 ± 0.9
ε (%)	0.6 ± 0.1	1.0 ± 0.1
HV	22.6 ± 0.2	20.4 ± 0.7

## Data Availability

The original contributions presented in the study are included in the article/supplementary material, further inquiries can be directed to the corresponding author.

## Supplementary Materials

The following supporting information can be downloaded at: https://www.mdpi.com/article/10.3390/polym16223205/s1, Figure S1: Fracture surfaces of 1.0 D-A (a,b) and 0 D-A (c,d) resins after the three-point bending tests; Figure S2: Mass loss rate of the epoxy resin according to the D-A ratio; Figure S3: Mass loss of the epoxy resin according to its D-A ratio bonds after being immersed in acetone and dimethylformamide at ambient temperature for 24 h.
