# Supplementary material for: Novel Recycling of Epoxy Thermosets by Blending with Reversible Diels–Alder Epoxy Resin
Source: Polymers (Basel). 2024 Nov 19;16(22):3205. doi: 10.3390/polym16223205 (PMC11598711; doi:10.3390/polym16223205)
Supplement: Supplementary file 1 [file polymers-16-03205-s001.zip › polymers-3284839-supplementary.pdf]

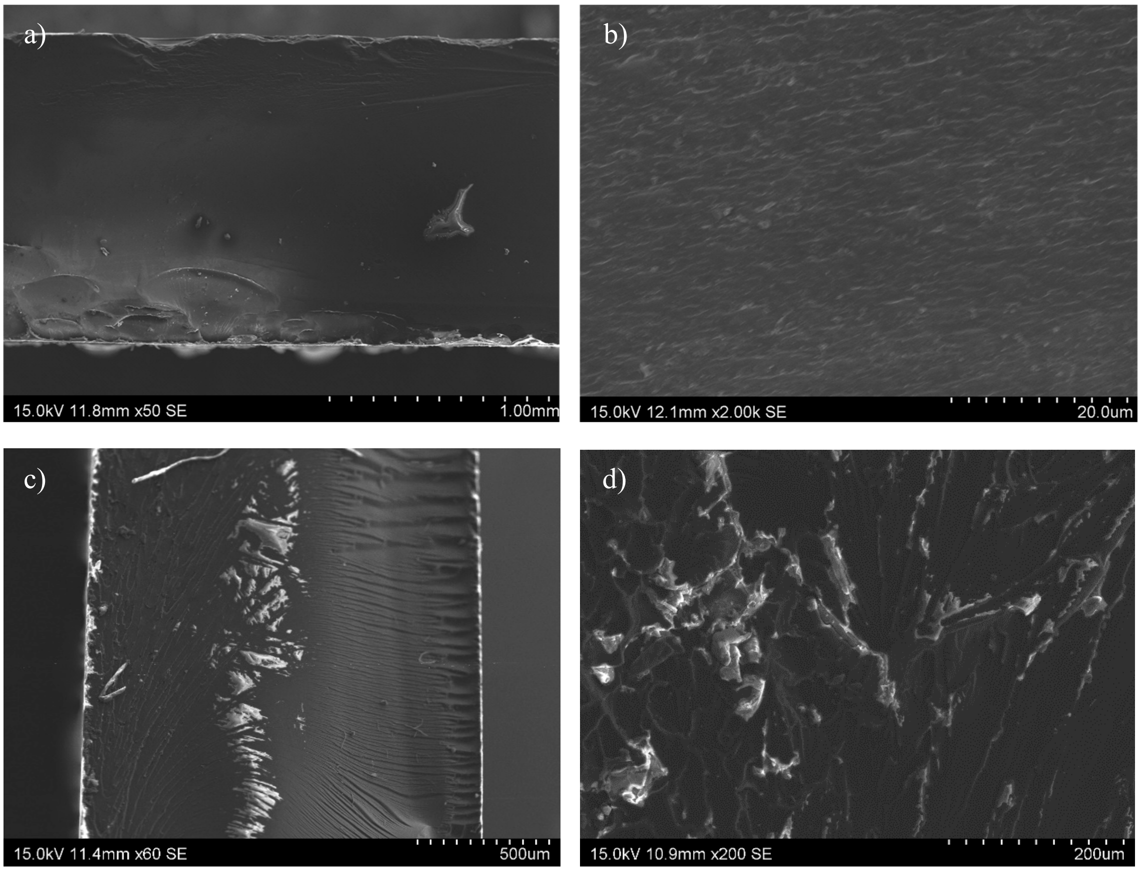

Figure S1. Fracture surfaces of the 1.0 D-A (a,b) and 0 D-A (c,d) resins after the three-point bending tests.

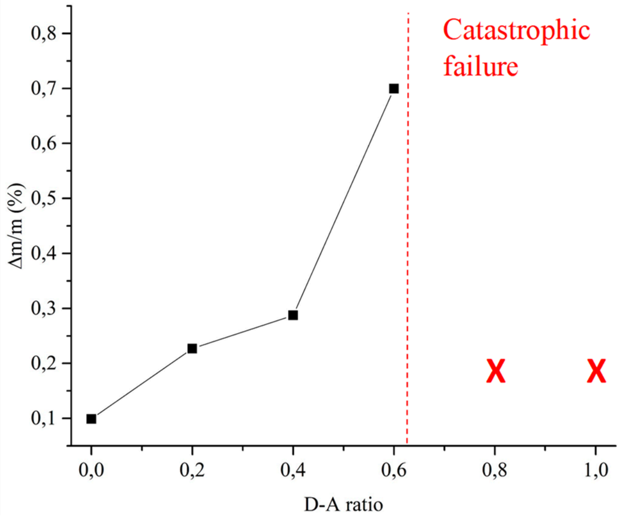

Figure S2. Mass loss rate of the epoxy resin according to the D-A ratio.

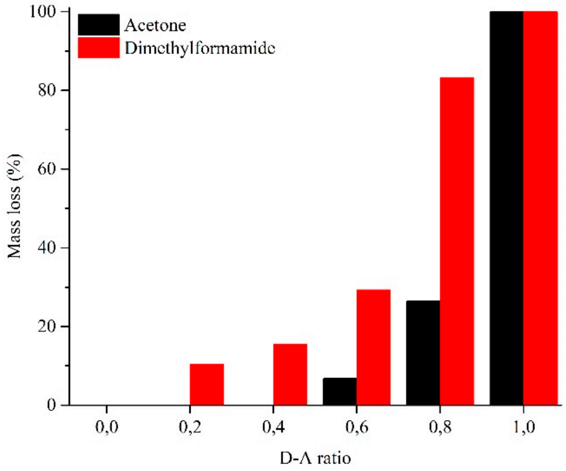

Figure S3. Mass loss of the epoxy resin according to its D-A ratio bonds after being immersed in acetone and dimethylformamide at ambient temperature for 24 hours.
